# Migration Experiences and Reported Sexual Behavior Among Young, Unmarried Female Migrants in Changzhou, China

**DOI:** 10.9745/GHSP-D-17-00068

**Published:** 2017-09-27

**Authors:** Zhanhong Zong, Wenjian Yang, Xiaoming Sun, Jingshu Mao, Xingyu Shu, Norman Hearst

**Affiliations:** a Institute of Public Administration, Hohai University, Nanjing, China.; b School of Economics, Nanjing University of Posts and Telecommunications, Nanjing, China.; c Department of Family and Community Medicine, University of California, San Francisco, CA, USA.

## Abstract

**Background:**

China has a large migrant population, including many young unmarried women. Little is known about their sexual behavior, contraceptive use, and risk of unintended pregnancy.

**Methods:**

475 unmarried female migrants aged 15–24, working in 1 of 6 factories in 2 districts of Changzhou city, completed an anonymous self-administered questionnaire in May 2012 on demographic characteristics, work and living situation, and health. We examined demographic and migration experience predictors of sexual and contraceptive behavior using bivariate and multivariate regressions.

**Results:**

30.1% of the respondents were sexually experienced, with the average age at first sex of 19 years (standard deviation=3). 37.8% reported using contraception at first sex, 58.0% reported using consistent contraception during the past year, and 28.0% reported having at least 1 unintended pregnancy with all unintended pregnancies resulting in abortion. Those who had had at least 1 abortion reported having on average 1.6 abortions [SD=1] in total. Migrating with a boyfriend and changing jobs fewer times were associated with being sexually experienced. Younger age, less education, and changing jobs more times were associated with inconsistent contraceptive use.

**Conclusion:**

These findings demonstrate there is an unmet need for reproductive health education and services where these women work as well as in their hometown communities. This education must begin early to reach young women before they migrate.

## Abstract (precis)

30% reported being sexually experienced, but only 38% reported using contraception at first sex and 58% consistently over the past year, leading to many unintended pregnancies and abortions. These findings document an unmet need for reproductive health education and services for young, unmarried female migrants in urban China.

## INTRODUCTION

China has experienced rapid economic development since the early 1980s, which has produced massive internal migration to meet the labor needs of the industrialized urban areas of eastern China. The migrant population in China has progressively increased since the 1980s, from 6.5 million in 1982 to 48.4 million in 1995, 144 million in 2000, and 221 million in 2010, with most migrants from rural areas.^1^^–^^3^ Under the current *hukou* (household registration) system, migrants leave their relatively poor and underdeveloped hometown, which remains their place of household registration, and move to work in other areas without official approval.^4^

Health care for migrants is not covered by the public service system because services are provided only for people with official local household registration.^5^ Private health services are limited and expensive. Meeting the needs of this large and increasing migrant population has thus challenged the Chinese health care system.^6^^–^^9^ The majority of the migrant population is young (about half are between the ages of 15 and 24 years), but few studies have focused on young migrants' health.^5^^,^^10^^,^^11^

A public system of family planning clinics provides reproductive health services including free contraceptives and abortion but only for married couples of childbearing age. Although half of migrant women are under age 25 and unmarried,^12^ there are no government programs providing reproductive health services for unmarried young women, resulting in a large gap between available services and needs.^13^ There is little sexual education in schools and sometimes not even within the family because of conservative social norms that unmarried young women should not be having sex.^14^

Although half of migrant women in China are under 25 and unmarried, there are no government programs providing reproductive health services for unmarried young women.

Traditional Chinese values prohibiting premarital sex for women are often not observed. No studies in China have directly compared sexual activity of young migrant women in urban areas with their non-migrant counterparts, but there are reasons to think migrant women may be at higher risk. One study found that 60.8% of unmarried male migrants and 40.2% of unmarried female migrants aged 15–24 were sexually experienced.^15^ Another study showed that a greater percentage of rural-to-rural migrants aged 15–24 were sexually active than non-migrants of the same age in rural areas (53.5% vs. 47.4%, respectively).^16^ A study of children of adult migrant workers found that more middle school students of the migrant workers (average age, 14 years) were sexually active than non-migrant adolescents of the same age (7.2% vs. 4.5%, respectively).^14^ Other studies have shown a number of factors to be associated with sexual risk behavior, including gender, age, educational level, cultural background, rural vs. urban residence, and income level.^15^^–^^21^ A qualitative study found that female migrants far from their family may feel lonely and regard sex with a partner as a reward for companionship.^22^

Condom use may be lower among migrant adolescents than non-migrants of the same age (52.7% vs. 65.7%, respectively, in one study^15^). In one study, 21% of sexually active young migrants reported never using any contraception,^16^ and in another study 34.9% had unprotected sex in the last 3 months.^23^ Reasons for non-use of contraception among young migrants included believing it isn't necessary with their boyfriends or girlfriends.^19^ Many young women using the rhythm method could not compute the “safe” period correctly and had poor sexual and contraceptive knowledge.^12^^,^^22^^,^^24^^,^^25^

While adolescence and young adulthood are relatively healthy times of life, they can also be a period of social and health risks from unsafe sexual behavior, including sexually transmitted infections (STIs), unintended pregnancy, and abortion.^26^ One study of young migrants aged 15–24 found that 8.7% of female migrants had STIs and 47.4% of those sexually active had had an unintended pregnancy.^15^^,^^23^ Another study of young migrant women seeking abortion showed that 38.7% had had a previous abortion, 17.5% had never used condoms, and 9.7% had STIs.^27^

Although these studies indicate that young female migrants may be at reproductive health risk, little is known about what aspects of the migration experience are risk factors or which women may be at higher risk. This study examined sexual behavior of unmarried young female migrants working in factories in Changzhou, China, to determine patterns and predictors of risk, with particular attention to the migration experience.

Young female migrants in China may be at greater reproductive health risk than their non-migrant counterparts, but little is known about their migration experience and other risk factors.

## METHODS

### Setting

This cross-sectional, questionnaire-based study was conducted in 2 Economic Development Areas (zones designated by local government for building factories) in 2 districts of Changzhou city with large migrant populations. Changzhou is typical of the many rapidly developing cities in eastern China where the local population alone cannot meet the great demand for labor and a large number of young people, mostly from rural areas, move to the city for work. In 2010, the 6th Population Census showed 1.48 million migrants, including 0.38 million females aged 16–24 years, contributing to Changzhou's total population of 4.59 million (http://www.cztjj.gov.cn/class/OEJCMFCP). Most work in factories is located in Economic Development Areas far from the city center. As strangers to the city working long hours, they usually spend most of their time in factories and nearby worker quarters.^28^

### Sampling

Data for this study were gathered in May 2012 as part of the baseline measure of a wellness program aiming to improve migrant workers' health and rights in Changzhou. Purposive sampling was used to select 6 factories, each producing different products (such as underwear, electronic elements, outdoor equipment, and trains) from the 2 Economic Development Areas, seeking a representative sample of migrant workers. In each factory, the sampling fraction was about one-fifth, yielding 200–300 migrant workers through a systematic sample based on workers' ID numbers. In total, 1,665 respondents aged 16–48 years were sampled. This analysis included 475 unmarried females aged 16–24 years old who were a subset of the 1,110 female respondents.

### Instrument

A self-administered closed-ended questionnaire was pretested with 20 migrant workers in a sampled factory. We interviewed these respondents after administering the questionnaire to verify that they understood the questions; these respondents were then excluded from the formal survey. The instrument collected data on demographic characteristics, work situation, living situation, and health.

### Methods of Administration

Eighteen trained fieldworkers conducted the survey, with 3 assigned to each sampled factory. Permission was obtained from management of the sampled factories, and investigators conducted the survey for 1 day in each factory. A survey coordinator at each factory provided a list of sampled workers to workshop supervisors. Factories have infirmaries that provide basic first aid, and supervisors asked sampled workers to go to the infirmary for an interview about migrants at the end of their work shift if they were willing. Investigators explained clearly what would be asked when the women arrived. They were reminded of anonymity and the voluntary nature of the study and signed a consent form that was not attached to the anonymous questionnaire. Refusals were rare (as is often the case for survey research by scientific research institutions in China) and not recorded. The questionnaires were distributed to 8–10 migrant workers at a time by a fieldworker. The respondents took approximately 20 minutes to complete the self-administered questionnaire. Afterwards, participants received a gift valued at US$1.50.

### Independent Variables

We analyzed 2 main groups of independent variables: those related to demographic characteristics and those related to the migration experience. For the demographic characteristics, age was divided into 3 categories: 16–18 years, 19–21 years, and 22–24 years; education level was coded into 4 categories: elementary school, middle school, high school, and college; ethnic group was coded Han or Minority; only child in her family was coded yes or no; and hometown was coded rural or urban. For the migration experience variables, age at first migration was coded into 4 categories: 13–15 years, 16–18 years, 19–21 years, and 22–24 years. (Labor laws in China require women to be 16 years old; those migrating at younger ages either did not work right away or did so illegally.) Geographic spread was coded as originating in inner Jiangsu province (outside Changzhou) or another province; migrating with a boyfriend was coded yes or no; and number of jobs worked in different factories since migration was recorded as a number.

### Dependent Variables

Regarding sexual behavior, 3 dependent variables were considered. The first was a yes/no question as to whether the respondent had ever had sex. Additional questions asked of women who were sexually experienced included whether they always used contraception with sex in the past year. To be considered “consistent,” respondents had to answer yes to both of the following questions: “Did you use a contraceptive method at sexual behavior every time in the last year?” and “Did you use contraception at last sex?” The third question was “Did you ever have an unintended pregnancy?” If the answer was yes, they were asked, “How many abortions have you had in your lifetime?”

### Data Analysis

Data were analyzed with SPSS version 20.0. The bivariate associations between sexual experience and demographic characteristics and migration experiences were examined using the chi-square test. Associations between dichotomous and continuous variables were assessed using the *t* test. Associations between ordinal variables were analyzed using gamma correlation. We assessed independent predictors of dependent variables with forward stepwise logistic regression that retained predictors with *P*<.05 in the final multivariate model. Because age at migration and chronological age were highly correlated (gamma correlation 0.83), we did not include age at migration as a predictor in the multivariate analysis.

### Ethical Clearance

The Institutional Review Board at Nanjing College for Population Program Management approved the study protocol and survey instrument, and authorities of the local family planning commission also provided permission to conduct the study.

## RESULTS

### Demographic and Migration Characteristics

The sample consisted of 475 unmarried female migrants, of whom 59.4% were aged 16–21 years, 90.5% had at least a middle school education, 20.1% were the only child of their parents, 6.9% were ethnic minorities, and 88.0% came from a rural area (Table 1). Regarding migration experiences, 86.8% left their hometown and started to work before 22 years of age, 68.8% were from provinces other than inner Jiangsu, 5.9% migrated together with a boyfriend, and the average number of jobs held since migration was 4 (standard deviation [SD]=3), with a maximum of 16.

**TABLE 1.** Demographic and Migration Characteristics of Sampled Migrant Female Workers, Changzhou, China, 2012 (N=475)

Characteristic	No. (%)
Age group, years	
16–18	112 (23.6)
19–21	170 (35.8)
22–24	193 (40.6)
Ethnic group	
Han	442 (93.1)
Minority	33 (6.9)
Education level	
Elementary	45 (9.5)
Middle school	193 (40.6)
High school	156 (32.8)
College	81 (17.1)
Hometown	
Rural	418 (88.0)
Urban	57 (12.0)
An only child	
Yes	105 (20.1)
No	370 (79.9)
Age at first migration, years	
13–15	49 (10.3)
16–18	233 (49.1)
19–21	130 (27.4)
2224	63 (13.3)
Area of origin	
Inner Jiangsu	148 (31.2)
Other provinces	327 (68.8)
Migrating with a boyfriend	
Yes	28 (5.9)
No	447 (94.1)
No. of jobs held, mean (standard deviation)	4 (3)

### Sexual Activity

Among the entire sample, 30.1% of the respondents were sexually experienced, and the average age at first sex was 19 years (SD=3), with a minimum age of 13 years (data not shown). Most sexually experienced young females (87.7%) had first sex with a boyfriend, and 72% of them first had sex after migration. Table 2 shows bivariate associations of being sexually experienced with demographic characteristics and migration experiences. Significant predictors of being sexually experienced included older age, minority ethnic status, lower educational level, being an only child, migrating with a boyfriend, and having held fewer jobs since migration.

**TABLE 2.** Bivariate Predictors of Being Sexually Experienced Among Young Female Migrant Workers, Changzhou, China, 2012

Characteristic	n/N (%)	*P* Value
Age group, years		<.001
16–18	21/112 (18.8)	
19–21	42/170 (24.7)	
22–24	80/193 (41.5)	
Ethnic		<.001
Han	121/442 (27.4)	
Minority	22/33 (66.7)	
Education level		<.001
Elementary	27/45 (60.0)	
Middle school	49/193 (25.4)	
High school	37/156 (23.7)	
College	30/81 (37.0)	
Hometown		.17
Rural	121/418 (28.9)	
Urban	22/57 (38.6)	
Only child in the family		.002
Yes	45/105 (42.9)	
No	98/370 (26.5)	
Age at first migration,^a^ years		.08
13–15	14/49 (28.6)	
16–18	71/233 (30.5)	
19–21	37/130 (28.5)	
22–24	21/63 (33.3)	
Geographic spread of migration		.52
Inner Jiangsu	41/148 (27.7)	
Other provinces	102/327 (31.2)	
Migrating with a boyfriend		<.001
Yes	19/28 (67.9)	
No	124/447 (27.7)	
No. of jobs held, mean, sexually experienced group, unexperienced group	3.7, 4.4	.009

^a^ Age at first migration was not considered in the logistic models because its gamma correlation with age group was 0.83.

30% of the respondents were sexually experienced.

Table 3 shows significant independent demographic predicators of being sexually experienced in multivariate analysis, including older age, minority ethnic status, and being the only child in a family. As for migration experience, women migrating with their boyfriends were more likely to be sexually experienced than those migrating alone, and migrants having held more jobs since migrating were less likely to be sexually experienced.

**TABLE 3.** Multivariate Predictors of Being Sexually Experienced and of Inconsistent Contraceptive Use Among Young Female Migrant Workers, Changzhou, China, 2012 (N=143 Sexually Experienced Females)

Characteristic	Being Sexually Experienced			Inconsistent Contraceptive Use		
	OR	95% CI	*P* Value	OR	95% CI	*P* Value
Age group, years			<.001			.03
16–18	0.21	0.11, 0.41	<.001	4.32	1.21, 15.36	.02
19–21	0.45	0.27, 0.75	.002	3.55	1.18, 10.69	.02
22–24	1			1		
Education level			NS			.049
Elementary				4.15	1.11, 17.57	.046
Middle school				5.11	1.42, 43.32	.01
High school				1.74	0.46, 6.60	.41
College				1		
Ethnic group			<.001			NS
Han	0.19	0.08, 0.44				
Minority	1					
Only child in the family			.02			NS
Yes	1.8	1.10, 2.94				
No	1					
Migrating with a boyfriend			.001			NS
Yes	4.91	2.00, 12.08				
No	1					
No. of jobs held since migrating^a^	0.84	0.77, 0.91	<.001	2.02	1.56, 2.62	<.001

Abbreviations: CI, confidence interval; NS, not significant; OR, odds ratio.

^a^ OR for number of jobs held is for each additional job held.

In multivariate analysis, significant predicators of being sexually experienced included older age, minority ethnicity, being the only child, migrating with boyfriend, and holding fewer jobs since migration.

### Contraceptive Use

At first sex, 37.8% of respondents reported using contraception: 55.6% used the “morning after pill,” and 44.4% condoms (data not shown). Among those who did not use contraception at first sex, the 2 main reasons stated were “knew nothing about contraception” and “did not know how to use contraception.”

About 38% of respondents reported using contraception at first sex and 58% reported using it consistently over the past year.

Among those who were sexually experienced, 58.0% reported using contraception consistently over the past year. The most common methods used for consistent contraceptive users were condoms (62.6%) and hormonal pills (10.7%). Some women who used contraception consistently (23%) did not always use the same method, often using morning-after pills as a remedial measure if no contraception was used at sex. Other methods such as spermicides, hormonal injections, and intrauterine devices were almost never used by these young unmarried women.

Bivariate predicators of not using contraception consistently were lower educational level (44% with elementary school education reported not using contraception consistently compared with 57.1% with middle school education, 37.8% with high school education, and 20% with college education; *P*=.01); younger age at first migration (49.4% of those migrating at age 16–18 reported not using contraception consistently compared with 40.5% of those migrating at age 19–21 and 14.3% of those migrating at age 22–24; *P*=.01); and having held more jobs since migration (mean number of jobs with consistent contraceptive use 2.5 [SD=2] compared with mean for others 5 [SD=3]; *P*<.001). Other demographic predictors not significantly associated with consistent use of contraception included ethnic group, being the only child in the family, urban vs. rural hometown, province of origin, and migrating with a boyfriend.

Table 3 shows the significant predictors of inconsistent use of contraception in multivariate analysis. Older women had lower risk of inconsistent use of contraception, and those with middle school education reported inconsistent use to the greatest extent. Regarding migration experiences, the only significant predictor in the multivariate logistic model was that women who had held more jobs were less likely to use contraception consistently.

Significant predictors of inconsistent contraceptive use in multivariate analysis included age and holding more jobs since migration.

### Pregnancy and Abortion

Among the respondents who reported being sexually experienced, 28.0% reported having had an unintended pregnancy (data not shown). All of these pregnancies resulted in abortion. This is not surprising because most pregnancies among unmarried women in China result in abortion,^12^^,^^19^ and any women who might be exceptions probably would not continue working in these factories. The average number of abortions among women who had had at least 1 abortion was 1.6 (SD=1). Reasons reported for the last unintended pregnancy were non-use of contraception (75%) and contraceptive failure (25%). Among sexually experienced young females, 30.1% said they knew how condoms work, 38.5% knew about emergency morning-after pills after having unprotected sex, and 51.7% agreed that abortion is harmful to women's health. These percentages were not significantly different from women who were not sexually experienced.

28% of those who were sexually experienced reported having an unintended pregnancy, all resulting in abortion.

The only demographic characteristics that were significantly associated with unintended pregnancy in bivariate analysis were age group and educational level. In a logistic model including these 2 predictors, younger age (16–18, odds ratio [OR]=3.62, confidence interval [CI]=1.24 to 10.54; 19–21, OR=2.56, CI=1.01 to 5.51; index category, 22–24 years) and lower education level (elementary, OR=3.75, CI–1.01 to 14.71; middle school, OR=3.06, CI=1.01 to 10.56; high school, OR=0.91, CI=0.22 to 3.77; index category, college) were associated with unintended pregnancy. The migration experience variables examined had no significant association with unintended pregnancy.

Significant predicators of unintended pregnancy were age and education.

## DISCUSSION

This study of young, unmarried female migrants aged 16–24 in Changzhou, China, shows that a substantial proportion (30.1%) reported being sexually experienced. Of these, only about half report using contraceptives consistently, usually condoms. Many (28%) who are sexually experienced have had an unintended pregnancy. Although more report consistent contraceptive use in the past year than at first sex, many of these unmarried young female migrants continue to be exposed to the risk of unintended pregnancy.

Our findings show that a substantial proportion of young, unmarried female migrants in an urban area of China are sexually experienced, but only about half of them use contraception consistently.

The proportion of these women who report being sexually active seems relatively low compared with women internationally, although studies in other countries have not focused specifically on migrants.^29^^–^^31^ This may be due to many reasons, but traditional Chinese culture is probably a key one. The strongly conservative tradition of Confucianism emphasizes abstinence for unmarried women, with abstinence linked to personal and family honor.^17^ Nevertheless, many of these women are sexually active, and rates of sexual activity can only be expected to rise with modernization and globalization.

Findings from other studies about demographic predictors of sexual activity are consistent with our results regarding older age, lower educational level, and ethnic minority status.^17^^,^^21^ We also found associations not previously described between sexual activity and specific migration experiences, including age at first migration, whether migrating with a boyfriend, and number of jobs held since migrating.^13^^,^^14^^,^^17^^,^^18^

An interesting and novel finding of this study was the relation between number of jobs women have held since migrating and sexual behavior. For these women, having held more jobs is an indicator of economic instability. There is little upward job mobility, so changing jobs usually indicates that a woman was not doing well in her job or did not find work conditions to be acceptable. In our study, women in more stable job situations (i.e., who had worked fewer jobs) were more likely to be sexually active. Most women who had sex did so with a “boyfriend” (the Chinese term indicates an affective relationship). It is possible that women had more opportunity to establish such relationships when in a stable work situation or, conversely, that those involved in a romantic relationship were less likely to change jobs. On the other hand, women in less stable work situations (who had held a greater number of jobs) were less likely to use consistent contraception if they were sexually active. These findings should be further explored in future research.

### Limitations

As a cross-sectional survey, this study can only examine associations, not establish causality. Self-reported data may be subject to social desirability bias resulting in underreporting of premarital sex and overreporting of consistent contraceptive use. This study did not include a non-migrant comparison group for direct comparison. The variables we examined included only certain aspects of the migration experience. Results of this study in one city may not generalize to young female migrants elsewhere in China. But there are millions of young female migrants working in thousands of factories in eastern China who likely face similar problems, as may other young migrant women elsewhere in China and in other industrializing countries.^12^^,^^14^^–^^15^^,^^32^^–^^34^ Workers who migrate internationally likely face additional challenges. Nevertheless, we believe this study provides useful information about a large, poorly studied population of young migrant women at risk for unintended pregnancy.

## CONCLUSION

Many young, unmarried migrant women in China report being sexually experienced but report not using contraception consistently, often leading to unintended pregnancies. While abortion is safe and legal in China, it is considered a failure of contraception rather than a good family planning strategy. It would be better to provide women with the education and reproductive health services they need to prevent unwanted pregnancies.

Women who are younger, who migrate at an early age, who are less educated, and who have less stable work situations are less likely to use contraception consistently, suggesting that young women need appropriate sexual education at an earlier age, before they migrate. After they migrate, many of these women still need more sexual education as well as friendly and accessible reproductive health care services. Unfortunately, they usually receive neither. These women could benefit from a national policy to support reproductive health and service provision for unmarried young people, as some have suggested for China,^12^ or from direct provision of reproductive health services in the workplace or in partnership with nearby health facilities, as some have advocated internationally.^35^^–^^37^ Schools and health care services need to do more to protect these vulnerable young women. Correct knowledge and timely, targeted services can make a difference.

Young women in China need appropriate sexual education at an early age, before they migrate to urban areas.

## Notes

**Peer Reviewed**

**First Published Online:** 2017 September 12
